# Improved PET/MRI attenuation correction in the pelvic region using a statistical decomposition method on T2-weighted images

**DOI:** 10.1186/s40658-020-00336-5

**Published:** 2020-11-23

**Authors:** Elin Wallstén, Jan Axelsson, Joakim Jonsson, Camilla Thellenberg Karlsson, Tufve Nyholm, Anne Larsson

**Affiliations:** 1 Department of Radiation Sciences, Radiation Physics, Umeå University, 901 85 Umeå, Sweden; 2 Department of Radiation Sciences, Oncology, Umeå University, 901 85 Umeå, Sweden

**Keywords:** PET-MRI, PET, Attenuation correction, Pelvis, Prostate cancer

## Abstract

**Background:**

Attenuation correction of PET/MRI is a remaining problem for whole-body PET/MRI. The statistical decomposition algorithm (SDA) is a probabilistic atlas-based method that calculates synthetic CTs from T2-weighted MRI scans. In this study, we evaluated the application of SDA for attenuation correction of PET images in the pelvic region.

**Materials and method:**

Twelve patients were retrospectively selected from an ongoing prostate cancer research study. The patients had same-day scans of [11C]acetate PET/MRI and CT. The CT images were non-rigidly registered to the PET/MRI geometry, and PET images were reconstructed with attenuation correction employing CT, SDA-generated CT, and the built-in Dixon sequence-based method of the scanner. The PET images reconstructed using CT-based attenuation correction were used as ground truth.

**Results:**

The mean whole-image PET uptake error was reduced from − 5.4% for Dixon-PET to − 0.9% for SDA-PET. The prostate standardized uptake value (SUV) quantification error was significantly reduced from − 5.6% for Dixon-PET to − 2.3% for SDA-PET.

**Conclusion:**

Attenuation correction with SDA improves quantification of PET/MR images in the pelvic region compared to the Dixon-based method.

## Background

Correct quantification of radiotracer activity in PET is of importance when monitoring cancer treatment response. PET quantification relies on accurate attenuation measures, which is a remaining challenge with PET/MRI. In PET/CT systems, the attenuation map for 511 keV PET photons is calculated from the CT image, typically with a bilinear equation [1]. MR images are based on proton spin relaxation and contain no information about attenuation, which is caused by electron interactions. This causes difficulties using MR images for calculation of attenuation maps and leads to quantification errors in PET images.

The present model for attenuation correction (AC) of PET images of the body on the SIGNA PET/MRI (GE Healthcare, USA) is based on the 2-echo Dixon MRI sequence that produces images of water and fat separately. These images are used for segmentation into soft tissue, fat, lung and air, which are translated to attenuation coefficient values [2]. These attenuation coefficients will represent a populational mean rather than the individual variations between patients. The major problem with this method is however that bone of different densities is misclassified as fat, which leads to underestimated attenuation coefficients of bone and therefore also an underestimation of the PET tracer uptake.

Several approaches have been proposed to include bone information in the attenuation maps for PET/MRI. One method is implemented on the Biograph mCT (Siemens Healthcare, Germany), where segmentations from the Dixon sequence are combined with a model-based bone segmentation algorithm that adds bone information to the attenuation map [3]. In brain imaging, quantification has been improved with segmentation of ultra-short echo time [4] or zero echo time (ZTE) [5] MR images, and with atlas-based methods [6]. Recently, deep convolutional neural networks (CNNs) have been evaluated with promising results [7]. In the pelvic region, the atlas-based methods have more challenges with larger differences in inter-individual patient anatomy [8], but methods utilizing machine learning and CNNs have shown more success. Qian et al. proposed a fuzzy clustering method with Dixon MRI as input [9]. For CNNs, Leynes et al. used ZTE and Dixon MRI as input to generate pseudo CTs, also called synthetic CTs (sCTs) [10], whereas Torrado-Carvajal et al. used only Dixon MRI as input [11]. Hwang et al. used only PET data (without any MRI input), with attenuation maps and PET images from maximum-likelihood reconstructions of activity and attenuation (MLAA) as input to a CNN [12], for generation of whole-body CT derived AC as output. Finally, Bradshaw et al. trained a network with only diagnostic MRI (T2 and T1 LAVA Flex water only) as input, with a four-segment CT image as output [13].

In addition to the potential improvement of PET/MRI quantification, sCTs could also be used in radiotherapy, where MRI-only treatment-planning workflows would be preferable to avoid registration errors between MRI and CT and to streamline the workflow by avoiding CT acquisition [14]. The major difference between these applications is the photon energies, where the 511 keV photons of PET are more sensitive to attenuation differences than radiotherapy photons, with mean energies of typically 2–5 MeV (6–15 MeV maximum energy). It is therefore expected that sCT performs better in radiotherapy planning than for PET AC.

In this project, we evaluate a statistical decomposition algorithm (SDA) [15] for calculation of sCTs from T2-weighted MRI scans. The method is developed for radiotherapy dose planning and is a commercially available CE-marked product. We apply this algorithm to calculate attenuation maps for [^11^C]acetate PET/MRI images. The attenuation corrected PET images are validated against AC based on CT and the method is also compared with the Dixon-based method on the PET/MRI scanner.

## Materials and methods

### Image acquisition

Twelve prostate cancer patients (mean age 72.3 years; range 64–78 years; mean weight 79.3 kg; range 71–95 kg) were selected retrospectively from a clinical research study (ClinicalTrials.gov ID NCT01962324). All patients underwent [11C]acetate PET/MRI and CT scans on the same day. Patients were included if they had a radiotherapy treatment planning with delineated regions for prostate, and one hotspot volume within the prostate, and saved PET raw data. Patients were excluded if they had implants in the scanned field of view (FOV).

#### PET/MRI data

A 35-min, static single-bed PET scan was acquired on a SIGNA PET/MRI scanner (GE Healthcare), commencing 12.6 ± 3.3 min after injection of a 431 ± 53-MBq bolus dose of [11C]acetate.

A 2-echo Dixon T1-weighted MRI sequence (Liver Acquisition with Volume Acceleration: LAVA-Flex) was acquired for built-in AC based on segmentation of fat and water with in-phase and out-phase images. The following parameters were used: repetition time 4.05 ms, echo time 1.12 ms and 2.23 ms, flip angle 5°, matrix size 258 × 258, pixel size 1.95 × 1.95 mm^2^, slice thickness 5.2 mm with a 2.6 mm overlap, and 120 slices in total. T2-weighted scans (fast spin echo, FSE) were acquired with repetition time 15 s, echo time varying between 96.2 and 102.8, flip angle 130°, matrix size 1024 × 1024, pixel size 0.44 × 0.44 mm^2^, slice thickness 2.5 mm, and 98–112 slices depending on patient anatomy.

#### CT data

CT images were acquired on a Brilliance Big Bore CT (Philips Healthcare) with tube voltage 120 kV, automatic exposure (204 ± 94 mAs), matrix size 512 × 512, pixel size 1.014 × 1.014 mm^2^, and slice thickness 2 mm.

#### Statistical decomposition algorithm

The SDA (15) can be described as an atlas-based technique where tissues are registered separately. It takes a T2-weighted MRI scan as input and returns the most probable CT representation as output. The SDA was utilized from the online software MriPlanner (Spectronic Medical AB, Sweden). The algorithm uses a template database consisting of 15 MRI and CT scans from the same individuals, pairwise registered using non-rigid registration, with delineated structures of the prostate, bladder, colon, bones, and subcutaneous fat.

The SDA can be divided into three steps. The first step is automated segmentation of the incoming MRI. In this step, the template MRIs are registered to the incoming MRI, and the deformation fields are applied on the template segmentations to generate a set of segmentation candidates. The final segmentation is calculated using a weighted voting-method based on machine learning [16] which propagates the candidates that best resembles the incoming MRI. The second step of the SDA is warping, where all templates are deformed to match the segmented structures. These deformation fields are used as initialization of a constrained non-rigid registration, and the new deformation fields are applied on the template CTs, generating a set of candidate sCTs. The third and last step is fusion of the candidate sCTs by calculating the weighted median CT value for each voxel. The weights are calculated with a machine learning method which promotes the HU values of the candidate sCTs that most accurately resembles the features of the incoming MRI.

### Data pre-processing

The CT scans were adapted to the PET/MRI space with registrations performed with the Elastix toolbox [17] implemented in MICE-Toolkit (NONPI Medical AB, Umeå, Sweden). CT images were registered to T2-weighted MR images with affine registration, followed by non-rigid bspline transform registration using a mutual information metric with bending energy penalty. Full elastix parameter files are provided in the supplementary material. After CT registration, CT and SDA-CT were re-sampled to the same matrix size as the synthetic CT generated as a step in the built-in AC (Dixon-CT). Air cavities can be expected to move between and during scans and were therefore replaced with the CT value of water (0 Hounsfield units, HU). The CT images did not cover the exact same field of view in axial direction as the Dixon-CT; some inferior slices were missing in the CT-FOV. To approximate these missing slices (on average 3.1 slices were missing), the closest slice on the CT was repeated. PET slices where CT data was missing were excluded from the analysis.

The outermost part of the skin was barely visible on the T2-weighted scans, and the out-of-phase images displayed blurred contours, which creates uncertainties in patient contour. If not corrected for, these uncertainties can propagate into the registered CT, since the T2-weighed scans are used for registration. The uncertainties were eliminated by adapting the outer contour for CT and SDA-CT to the same contour as the Dixon-CT. This was achieved by replacing voxel-values in CT and SDA-CT outside the outer contour of the Dixon-CT with the CT-value of air (HU − 1000), and add tissue with the CT value of water (0 HU) where the Dixon-CT was larger than the CT or SDA-CT.

### PET reconstruction

PET images were reconstructed with AC based on each of the three imaging methods (Dixon-PET, SDA-PET, and CT-PET) employing the PET recon toolbox (GE Healthcare), version MP26, release 30.4. Time-of-flight ordered-subset expectation-maximization (TOF-OSEM) was chosen to reconstruct the data according to our clinical protocol: 2 iterations, 28 subsets, 600 mm transaxial FOV, 25 cm axial FOV, matrix size 192 × 192, 89 slices, pixel size 3.125 × 3.125 mm, and slice thickness 2.78 mm. Attenuation information for CT and SDA-CT was fed into the PET recon toolbox as CT images, which were re-calculated into AC-maps (CT-AC and SDA-AC). Dixon-based AC maps (Dixon-AC) were also generated with the PET recon toolbox as a part of the reconstruction process. PET reconstructions were performed with truncation completion activated, which means that missing parts of the patient’s outer contour were estimated from the PET images [18].

### Analysis

The accuracy of Dixon-PET and SDA-PET were calculated over the whole FOV, using only the slices that were present in all AC-maps. CT-PET was considered as the ground truth. Voxels were segmented into the bone and soft tissue based on the attenuation map from the CT, where all voxels with attenuation coefficients higher than 0.102 cm^−1^ were segmented as bone. Images of percentage differences were calculated. From these difference images, values of mean error, mean absolute error (MAE), standard deviation (SD), and root mean square error (RMSE) were calculated for the whole image and separately for the above-segmented bone and soft tissue. The same statistical measures were calculated comparing Dixon-AC and SDA-AC with CT-AC as ground truth.

For visualization of the PET and AC error distribution, error maps averaged over all patients were calculated in a common space. One patient was manually selected as reference geometry, where the selection aimed for an average patient size. All error maps were then subject to a non-rigid registration to the geometry of this reference patient. The T2-weighed MRI of each patient was registered to the T2-weighted MRI of the reference patient with the same method as described for the CT to MRI registration in the data pre-processing section, and the deformations were applied on the error maps for each patient. The registered error maps were used for calculation of a mean error-map for all patients.

Lesion analyses were performed with volumes of interest (VOIs) drawn by specialists in oncology as a part of radiotherapy treatment planning. The prostate and a prostate-hotspot were compared regarding mean standardized uptake value (SUV) within the VOI. SUV error (%ΔSUV) was calculated for Dixon-PET and SDA-PET as a percentage difference relative to CT-PET. Statistical significance was tested with the Wilcoxon signed-rank test.

## Results

**Fig. 1:**
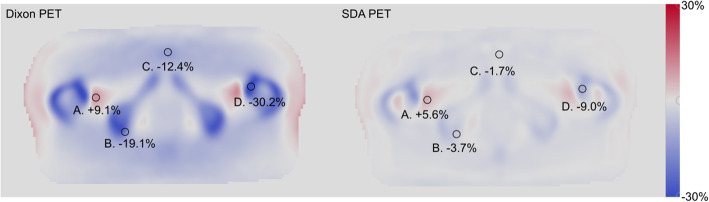
PET error maps, averaged over all patients. Regions A–D are samples of regions with large positive and negative errors. A–D are identically located in the two images

The Dixon-PET error map (Fig. 1) demonstrates the absence of bone attenuation information in this algorithm, where the activity inside and close to bone is underestimated, and some areas in between bone regions are overestimated. SDA-PET shows similar patterns but with considerably lower deviations. For example, region D (Fig. 1) is underestimated relative the CT-PET, with a deviation of -30% for Dixon-PET and -10% for SDA-PET. For the overestimated regions, region A (Fig. 1) displays a 9.1% error for Dixon-PET and a 5.6% error for SDA-PET.

**Fig. 2:**
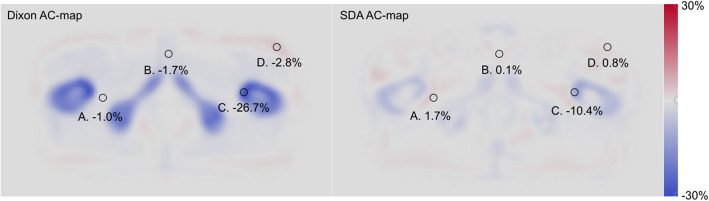
Attenuation coefficient error maps, averaged over all patients. Regions A–D are samples of regions with large positive and negative attenuation value errors. A–D are identically located in the two images

The absence of bone information is also very clear in the error maps for attenuation coefficients (Fig. 2), where a clear underestimation in the bone can be seen. The AC-map for SDA shows a smaller underestimation of bone attenuation than the Dixon method.

**Fig. 3:**
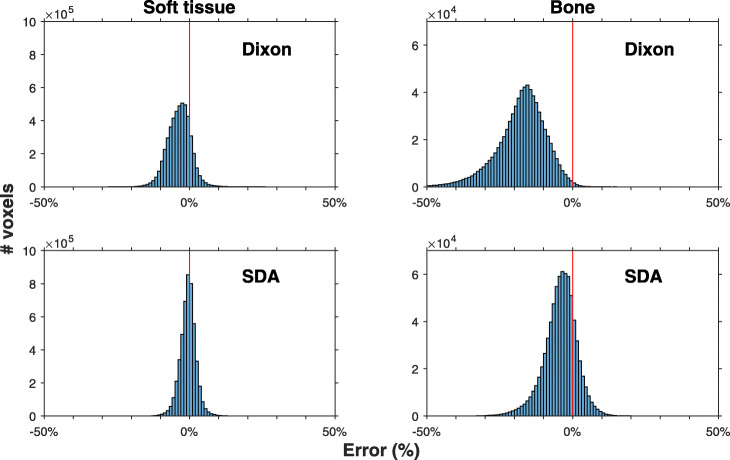
Histograms of PET uptake error for Dixon and SDA reconstructions, for voxels classified as soft tissue and bone. Soft tissue and bone were classified individually for each patient’s image. Vertical line indicates 0% error

Histograms of PET image voxel errors (Fig. 3) confirm that the errors for Dixon-PET are skew towards the negative side, whereas the histograms for SDA-PET are more symmetric and narrower. The error histogram for SDA-PET soft tissue is almost completely centred with a mean error of − 0.5%, compared to − 3.6% for Dixon-PET (Table 1). Corresponding mean errors for bone are − 4.2% (SDA-PET) and − 17.7% (Dixon-PET).

**Table 1:** Error statistics for PET images and AC-maps; mean values, standard deviations (SD) mean absolute error (MAE) and root mean square error (RMSE)

	PET				AC-map			
	Mean	SD	MAE	RMSE	Mean	SD	MAE	RMSE
Dixon whole volume	− 5.4%	6.7%	6.1%	8.6%	− 2.9%	6.3%	3.7%	6.9%
SDA whole volume	− 0.9%	3.5%	2.5%	3.6%	− 0.6%	3.6%	2.1%	3.6%
Dixon bone	− 17.7%	8.4%	17.7%	19.5%	− 16.3%	6.7%	16.3%	17.6%
SDA bone	− 4.2%	5.7%	5.4%	7.1%	− 4.4%	6.5%	5.3%	6.8%
Dixon soft tissue	− 3.6%	4.0%	4.3%	5.4%	− 0.9%	2.6%	1.8%	2.7%
SDA soft tissue	− 0.5%	2.7%	2.1%	2.8%	− 0.3%	2.4%	1.7%	2.4%

Mean values, standard deviations (SD), mean absolute error (MAE), and root mean square error (RMSE) for voxels classified as bone or soft tissue and for whole volume

**Fig. 4:**
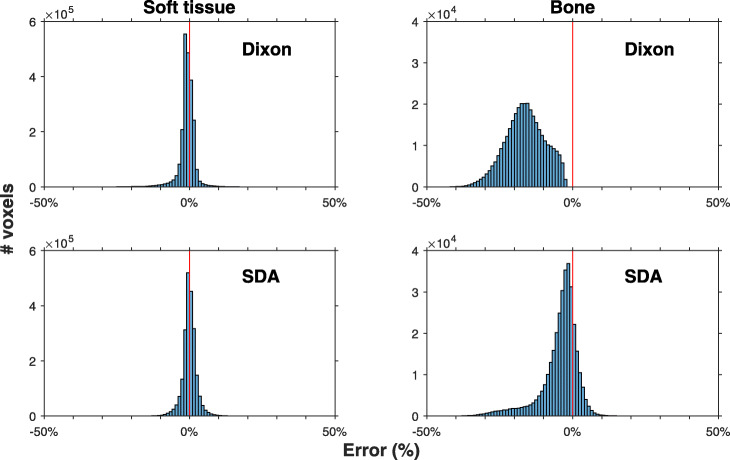
AC-map error histograms for Dixon and SDA reconstructions for voxels classified as soft tissue and bone. Soft tissue and bone were classified individually for each patient’s image. Vertical line indicates 0% error

For AC-maps, soft tissue histograms (Fig. 4) are narrow and centred around zero for both Dixon-AC (mean error − 0.9%) and SDA-AC (mean error − 0.3%). Bone histograms show more variability. The histogram for Dixon-AC (mean error − 16.3%) is underestimated in all bone voxels (all values < 0). The histogram for SDA-AC (mean error − 4.4%) on average also underestimates the attenuation coefficient, but also overestimates the value in some bone voxels.

**Fig. 5:**
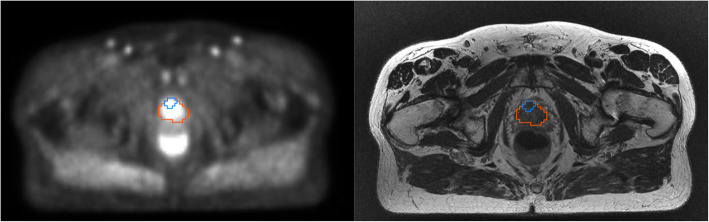
Lesions for one patient on PET and T2-weighted MRI. The prostate (red) and hotspot (blue) were drawn by specialists in oncology as a part of radiotherapy treatment planning, based on multiple MR sequences and PET images

**Fig. 6:**
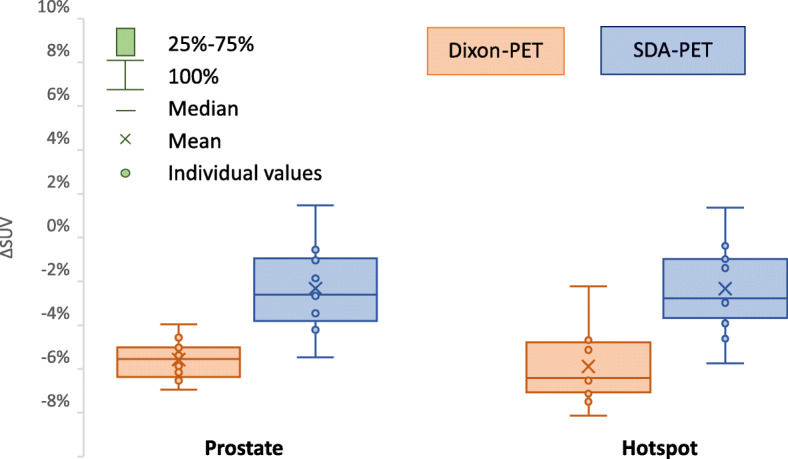
Box and whisker plots of errors in SUV (%ΔSUV) for prostate and hotspot lesions

The lesions in this study consisted of the prostate (mean volume ± SD; 47.7 ± 22.4 ml), including also one hotspot (3 ± 4.7 ml), which can be seen in Fig. 5. The uptake relative to CT-PET (%ΔSUV) is plotted in Fig. 6 for Dixon-PET and SDA-PET. The boxplots for both lesion types show similar behaviour, with significant lower errors for SDA-PET. The mean %ΔSUV for the prostate lesion was − 5.6% (Dixon-PET) and − 2.3% (SDA-PET), where the Wilcoxon signed-rank test showed a significant difference between the methods (*p* = 0.0024). For hotspot lesions, the mean values were − 5.9% (Dixon) and − 2.3% (SDA), also with a significant difference between methods (*p* = 0.00098).

## Discussion

We have evaluated SDA-generated synthetic CT (sCT) images for attenuation corrected PET reconstructions in the pelvic region and compared that with attenuation corrected PET reconstructions generated with CT images. The SDA calculates sCTs from T2-weighted MRI sequences with a probabilistic atlas-based technique, where the most probable CT representation for a given T2 image is calculated. The results are promising despite the fact that other atlas methods have had limited success in the pelvic region [8].

The error maps in Fig. 1 (PET) and Fig. 2 (AC) illustrates the distribution of errors. It is clear that attenuation coefficients and PET activity in bone and areas close to bone are most underestimated with the scanner’s built-in Dixon-method, and that the errors are substantially reduced with the SDA-method. A potential bone metastasis in position C in Fig. 1 would have a quantification error of − 19.1% with the Dixon method, which is reduced to − 3.7% using the SDA method. The attenuation coefficients are underestimated in the Dixon AC-map, which propagates to quantitation errors in the PET image.

Three previous studies have presented attenuation maps from MRI using neural networks, with ZTE and Dixon sequences as input in a method called ZeDD [10], with only Dixon sequences as input in a method called DIVIDE [11], and with diagnostic MRI (T2 and LAVA Flex water only) [13]. All studies show improvements compared with the Dixon-based method. Bradshaw et al. reported an improvement in whole volume RMSE from 11.6% (Dixon) to 4.9%, which can be compared to our reported RMSE improvement from 8.6% (Dixon) to 3.6% (SDA). PET-ZeDD improved RMSE in soft tissue from 6.10% (Dixon) to 2.85%, where we reported similar numbers with improvement in RMSE from 5.4% (Dixon) to 2.8% (Table 1). PET-DIVIDE reported a change in soft tissue absolute value recovery coefficients from 6.71% (Dixon) to 1.83%, which can be compared to our mean absolute error (MAE, Table 1) in soft tissue that decreased from 4.3% (Dixon) to 2.1%. The bone masks in this study were calculated on attenuation maps from CT with a threshold of 0.102 cm^−1^. The threshold was chosen to minimize the risk of misclassifying bone as soft tissue, and a higher threshold can be expected to yield higher errors in soft tissue since the largest errors are close to bone.

The main benefit of the SDA-method is that it only relies on T2-weighted images, which are commonly acquired for diagnostic purposes. This means that the scanning time does not have to be prolonged, since additional sequences are not required. This is also a benefit of the method by Bradshaw et al. [13] with only diagnostic MRI as input, and of the PET input only CNN method by Hwang et al. [12], even if absence of MRI derived information could risk missing valuable information. Another advantage with the current SDA method is that it is commercially available, which means that it can be readily implemented.

Studies that compare sCTs with CTs need to rely on registration, which entails some uncertainties also in this study. First, the registration in itself leads to uncertainties, even if the registrations were visually assessed to be accurate in the studied region. The largest registration uncertainties were found in the patient outer contour, where we chose to adapt all AC-maps to the outer contour for the Dixon method. This was done to minimize the uncertainties in the registration of the CT which was used as a gold standard. Another shortcoming related to registration is that bowel air can move between examinations. To minimize the effect, the voxel values of air pockets were replaced with HU 0 for water.

The reconstruction of the PET images in this study is conducted according to our clinical protocol; TOF-OSEM with 2 iterations and 28 subsets. It is possible that more iterations could have some effects on the results for the Dixon-PET, where the absence of bone information gives larger errors in the attenuation maps and affects the PET image globally [19]. The number of iteration is however in line with other studies [10–12], and our results are therefore comparable.

In this study, the patient group is limited to prostate cancer patients, a male population with a higher age than the average population. This age group can be expected to have lower HU values in bone, and it remains to evaluate if the results of this study are generally applicable to other age groups, and to female patients, or if the SDA algorithm may have to be adapted. Although the SDA captures bone, full HU recovery is not reached. It is hard to speculate about the reasons, but anatomical differences between this patient group and the patients in the template database could possibly be an explanation.

## Conclusion

We have evaluated a probabilistic atlas method for AC of pelvic PET images in PET/MRI scanners. The evaluated method improves quantification compared to the currently used Dixon-based method.

## Supplementary Information

**Additional file 1.** Parameters

## Data Availability

The datasets used and/or analysed during the current study are available from the corresponding author on reasonable request.

## Abbreviations

PET: Positron emission tomography
MRI: Magnetic resonance imaging
MR: Magnetic resonance
ZTE: Zero echo time
CNN: Convolutional neural networks
sCT: Synthetic computed tomography
MLAA: Maximum-likelihood reconstructions of activity and attenuation images
SDA: Statistical decomposition algorithm
FOV: Field of view
CT: Computed tomography
HU: Hounsfield unit
AC: Attenuation correction
TOF-OSEM: Time-of-flight ordered subset expectation maximization
VOI: Volume of interest
SUV: Standardized uptake value
MAE: Mean absolute error
SD: Standard deviation
RMSE: Root mean square error
