# Supplementary material for: Improved PET/MRI attenuation correction in the pelvic region using a statistical decomposition method on T2-weighted images
Source: EJNMMI Phys. 2020 Nov 23;7:68. doi: 10.1186/s40658-020-00336-5 (PMC7683750; doi:10.1186/s40658-020-00336-5)
Supplement: Supplementary file 1 — Additional file 1. Parameters [file 40658_2020_336_MOESM1_ESM.pdf]

Elastix parameter files

Rigid registration

```
(FixedInternalImagePixelType "float")
(MovingInternalImagePixelType "float")
(FixedImageDimension 3)
(MovingImageDimension 3)
(UseDirectionCosines "true")

// **************** Main Components **************************

(Registration "MultiResolutionRegistration")
(Interpolator "LinearInterpolator")
(ResampleInterpolator "FinalBSplineInterpolator")
(Resampler "DefaultResampler")
(FixedImagePyramid "FixedSmoothingImagePyramid")
(MovingImagePyramid "MovingSmoothingImagePyramid")
(Optimizer "AdaptiveStochasticGradientDescent")
(Transform "EulerTransform")
(Metric "AdvancedMattesMutualInformation")

// ***************** Transformation **************************

(AutomaticScalesEstimation "true")
(AutomaticTransformInitialization "true")
(AutomaticTransformInitializationMethod "GeometricalCenter")
(HowToCombineTransforms "Compose")

// ******************* Similarity measure *********************

(NumberOfHistogramBins 32)
(ErodeMask "false")

// ******************** Multiresolution **********************

(NumberOfResolutions 3)

// ******************* Optimizer ****************************

(MaximumNumberOfIterations 300)
(MaximumNumberOfSamplingAttempts 8.000000)
(RequiredRatioOfValidSamples 0.25)
(MaximumStepLength 4.0 2.0 1.0)

// **************** Image sampling **********************

(NumberOfSpatialSamples 2048)
(NewSamplesEveryIteration "true")
(ImageSampler "RandomCoordinate")
```

```
// ************* Interpolation and Resampling ****************

(FinalBSplineInterpolationOrder 3)
(DefaultPixelValue 0)
(ResultImagePixelType "float")
```

Affine registration

```
(FixedInternalImagePixelType "float")
(MovingInternalImagePixelType "float")
(FixedImageDimension 3)
(MovingImageDimension 3)
(UseDirectionCosines "true")

// **************** Main Components *************************

(Registration "MultiResolutionRegistration")
(Interpolator "LinearInterpolator")
(ResampleInterpolator "FinalBSplineInterpolator")
(Resampler "DefaultResampler")
(FixedImagePyramid "FixedSmoothingImagePyramid")
(MovingImagePyramid "MovingSmoothingImagePyramid")
(Optimizer "AdaptiveStochasticGradientDescent")
(Transform "AffineTransform")
(Metric "AdvancedMattesMutualInformation")

// ***************** Transformation *************************

(AutomaticScalesEstimation "true")
(AutomaticTransformInitialization "true")
(AutomaticTransformInitializationMethod "GeometricalCenter")
(HowToCombineTransforms "Compose")

// ******************* Similarity measure *********************

(NumberOfHistogramBins 32)
(ErodeMask "false")

// ******************** Multiresolution *********************

(NumberOfResolutions 3)

// ******************* Optimizer ****************************

(MaximumNumberOfIterations 300)
(MaximumNumberOfSamplingAttempts 8.000000)
(RequiredRatioOfValidSamples 0.25)
(MaximumStepLength 4.0 2.0 1.0)

// **************** Image sampling *********************

(NumberOfSpatialSamples 2048)
```

```
(NewSamplesEveryIteration "true")
(ImageSampler "RandomCoordinate")

// ************* Interpolation and Resampling ***************

(FinalBSplineInterpolationOrder 3)
(DefaultPixelValue 0)
(ResultImagePixelType "float")
```

Non-rigid registration

```
(FixedInternalImagePixelType "float")
(MovingInternalImagePixelType "float")
(FixedImageDimension 3)
(MovingImageDimension 3)
(UseDirectionCosines "true")

// **************** Main Components *************************

(Registration "MultiMetricMultiResolutionRegistration")
(Interpolator "LinearInterpolator")
(ResampleInterpolator "FinalBSplineInterpolator")
(Resampler "DefaultResampler")
(FixedImagePyramid "FixedSmoothingImagePyramid")
(MovingImagePyramid "MovingSmoothingImagePyramid")
(Optimizer "AdaptiveStochasticGradientDescent")
(Transform "BSplineTransform")
(Metric "AdvancedMattesMutualInformation"
"TransformBendingEnergyPenalty")
(Metric0Weight 0.5)
(Metric1Weight 0.5)

// ******************** Multiresolution *********************

(NumberOfResolutions 4)

// ***************** Transformation *************************

(FinalGridSpacingInPhysicalUnits 20)
(HowToCombineTransforms "Compose")

// ******************* Similarity measure ********************

(NumberOfHistogramBins 32)
(ErodeFixedMask "false")
(ErodeMovingMask "false")

// ******************* Optimizer ***************************

(MaximumNumberOfIterations 512 512 512 3064)
(MaximumNumberOfSamplingAttempts 8)
```

```
// **************** Image sampling **********************

(NumberOfSpatialSamples 2048)
(NewSamplesEveryIteration "true")
(ImageSampler "RandomCoordinate")

// ************* Interpolation and Resampling ****************

(FinalBSplineInterpolationOrder 3)
(DefaultPixelValue 0)
(ResultImagePixelType "float")
```
